# A Parent-of-Origin Effect Determines the Susceptibility of a Non-Informative F1 Population to *Trypanosoma cruzi* Infection *In Vivo*

**DOI:** 10.1371/journal.pone.0056347

**Published:** 2013-02-11

**Authors:** Grace K. Silva, Larissa D. Cunha, Catarina V. Horta, Alexandre L. N. Silva, Fredy R. S. Gutierrez, João S. Silva, Dario S. Zamboni

**Affiliations:** 1 Department of Cell Biology, University of São Paulo, Medical School Ribeirão Preto, FMRP/USP, Ribeirão Preto, São Paulo, Brazil; 2 Department of Biochemistry and Immunology, University of São Paulo, Medical School Ribeirão Preto, FMRP/USP, Ribeirão Preto, São Paulo, Brazil; Instituto de Ciências Biomédicas/Universidade de São Paulo – USP, Brazil

## Abstract

The development of Chagas disease is determined by a complex interaction between the genetic traits of both the protozoan parasite, *T. cruzi*, and the infected host. This process is regulated by multiple genes that control different aspects of the host-parasite interaction. While determination of the relevant genes in humans is extremely difficult, it is feasible to use inbred mouse strains to determine the genes and *loci* responsible for host resistance to infection. In this study, we investigated the susceptibility of several inbred mouse strains to infection with the highly virulent Y strain of *T. cruzi* and found a considerable difference in susceptibility between A/J and C57BL/6 mice. We explored the differences between these two mouse strains and found that the A/J strain presented higher mortality, exacerbated and uncontrolled parasitemia and distinct histopathology in the target organs, which were associated with a higher parasite burden and more extensive tissue lesions. We then employed a genetic approach to assess the pattern of inheritance of the resistance phenotype in an F1 population and detected a strong parent-of-origin effect determining the susceptibility of the F1 male mice. This effect is unlikely to result from imprinted genes because the inheritance of this susceptibility was affected by the direction of the parental crossing. Collectively, our genetic approach of using the F1 population suggests that genes contained in the murine chromosome X contribute to the natural resistance against *T. cruzi* infection. Future linkage studies may reveal the locus and genes participating on the host resistance process reported herein.

## Introduction

Human resistance to infectious diseases is usually regulated by multiple genes that control different aspects of the host-parasite relationship [1], [2]. Although it is difficult to achieve, the identification of such genes in humans is pivotal to understanding the essential processes leading to infection control. Murine models of experimental infection have facilitated the mapping of the genes because there is a high incidence of gene orthology between humans and mice. Inbred mouse strains that differ in their susceptibility to a given pathogen support the mapping of *loci* and genes that regulate resistance by permitting the analysis of segregation patterns in informative populations [3], [4]. Thus, the identification and detailed description of the variations in susceptibility to infection among inbred mouse strains are essential steps for developing successful models using forward genetic approaches to identify host factors that increase resistance to infectious diseases.

Chagas disease is caused by the intracellular parasite, *Trypanosoma cruzi*, and affects more than 10 million people in the world, mostly in Latin America [5], [6]. During the development of the disease, two distinct phases may be evident. An initial acute phase occurs when the parasite starts to replicate intracellularly; this phase is usually characterized by the presence of trypomastigote forms of the parasites in the blood and may lead to nonspecific symptoms such as fever and occasionally swelling and pain at the infection site. A chronic phase of the disease develops in approximately 30% of the infected individuals after years or decades without symptoms. This chronic phase is associated with a strong inflammatory response, which is induced by the presence of the parasites in the muscle tissues of different organs, particularly the heart and the digestive tract [7]. Although these distinct phases of the disease can be recognized in infected individuals, the development and manifestation of each phase are highly variable and depend on both the parasite strain and the host immune system. It is therefore well accepted that a combination of genetic components from the host and the parasite directly contribute to the outcome of the disease and its overall symptomology. Unfortunately, genetic manipulation of the *T. cruzi* parasites is challenging and has not been effectively achieved in the laboratory [8], [9]. Likewise, sexual crossing of *T. cruzi* parasites seems to be a rare phenomenon [10], [11]. These characteristics have impeded the investigation of *T. cruzi* genetic traits related to the pathogenesis of Chagas disease. Nonetheless, the use of the forward genetic approach in experimental models of infection may facilitate the identification of host genes responsible for disease development and for host resistance to infection [12].

In depth forward genetics with the African parasite, *T. congolense,* a main etiological agent of trypanosomiasis in African livestock, led to the identification of important host loci contributing to the control of infection by this parasite [3], [13], [14], [15], [16]. Although quantitative trait loci (QTL) regulating host resistance to *T. congolense,* which belongs to the *T. brucei* complex, have been identified, the scenario for *T. cruzi* is considerably different. *Trypanosoma* comprises a varied genus, with species that present wide differences in their replication sites, intracellular fate and pathogenesis in mammalian hosts [17]. Nevertheless, an outcross of susceptible parental mouse strains, C57BL/6 and DBA/2, was employed to demonstrate the existence of susceptibility *loci* on chromosomes 5, 13 and 17 that would lead to mouse resistance against the Tulahuen strain of *T. cruzi* [18]. Moreover, these studies suggested that a differential transcription of certain genes accounts for the differences in the susceptibility to the Tulahuen strain of *T. cruzi* [18]. Importantly, the Tulahuen strain, which belongs to the *T. cruzi* VI group, shows several differences compared to other widely used strains of the parasite, including the strains CL and Y, which belongs to the *T. cruzi* II group [19], [20], [21]. For instance, whereas C57BL/6 mice are susceptible to the Tulahuen strain, these inbred mice are resistant to the Y strain of *T. cruzi* [18], which is widely used for immunological investigation. The Y strain is highly virulent, leading to high peaks of parasitemia and inducing mortality in certain experimental models of infection [22], [23], [24]. Nonetheless, infection of certain inbred mice with the Y strain of *T. cruzi* results in an acute infection that is effectively controlled by a competent host. These features support the use of the Y strain of *T. cruzi* as a potentially valuable model to explore initial host factors that determine the outcome of the disease, which is intrinsically related to disease progression.

In this work, we evaluated the susceptibility of different inbred mouse strains to infection with the Y strain of *T. cruzi* and found a substantial difference in susceptibility between A/J and C57BL/6 mice, two strains that have been explored in detail. We employed a genetic approach to understand pathogenesis by investigating the pattern of inheritance of the resistance phenotype in an F1 population. Surprisingly, we detected a strong parent-of-origin effect determining the susceptibility of the F1 male mice. This effect could not be readily explained by gene imprinting because it depended on the direction of the parental crossing. Therefore, our data obtained with a non-informative F1 population suggested the existence of one or more *loci* contained in the murine chromosome X that contribute to the natural resistance to *T. cruzi* infection.

## Materials and Methods

### Mouse strains, parasites and infection

Ten- to twelve-week-old mice were used for the infection experiments. A/J, BALB/c, C3H/HePas, C57BL/6, DBA, F1(AXB) and F1(BXA) mice were maintained and bred in our institutional animal facilities. The mice were infected i.p. with 10^3^ blood-derived trypomastigotes from the Y strain of *T. cruzi*. Mouse parasitemia was monitored periodically by microscopic analysis of 5 μl blood samples drawn from the tail vein. Survival rates were determined by daily inspection of the cages. The mice were cared for according to the institutional guidelines on ethics in animal experiments (approved by the Comissão de Ética em Experimentação Animal da Faculdade de Medicina de Ribeirão Preto/CETEA protocol number 097/2010).

### Bone marrow-derived macrophage preparation, infection and analysis

Bone marrow-derived macrophages (BMDMs) were obtained as previously described [25]. Briefly, bone marrow cells were harvested from mouse femurs and differentiated with RPMI 1640 containing 20% fetal bovine serum and 30% L-929 cell-conditioned medium. Cells were cultures at 37°C in a 5% CO_2_ incubator. After seven days in culture, the BMDMs were harvested and seeded one day prior to infection on cover glass into wells (on 24 well plates) holding RPMI 1640 media, 10% FBS and 5% LCCM. The cells were infected at an MOI of 5 with trypomastigotes liberated from an LLC-MK2 cell culture. At 48 hours postinfection, the cells were fixed with methanol, stained with Giemsa and analyzed using a Leica DMI 4000B microscope. The number of amastigotes was estimated for 100 infected cells per cover glass, and the frequency of infection was compared among 6 replicates.

### Histopathological analysis

The mice were euthanized 15 days after infection, when samples of serum, cardiac muscle and liver were collected for biochemical and histopathological analysis. The tissue samples for the histological analysis were fixed in neutral 10% formalin, embedded in paraffin, sectioned (5 mm thick), hematoxylin–eosin stained, and examined by light microscopy. The cell density was estimated with ImageTool 2.0 software (University of Texas Health Science Center, San Antonio, TX, USA). The amounts of enzyme creatine kinase (CK-MB) and alanine aminotransferase (ALT) were quantified from the sera of these animals using a Labtest Kit. To determine the parasite concentration in the livers and the hearts of the infected mice, the tissue samples were minced, and DNA was extracted and purified using QIAmp DNA (Qiagen) according to the manufacturer's instructions. Quantitative PCR was performed using SYBR (Invitrogen) and a ‘StepOnePlus’ thermocycler (Applied Biosystems). The primers used were TCZ-F 5′-GCT CTT GCC CAC AMG GGT GC-3′ and TCZ-R 5′-CCAAGCAGCGGATAGTTCAGG-3′, which amplify a 195-bp sequence present in the genome of Y strain of *T. cruzi*, known as “satellite” DNA. The PCR conditions were previously described [26]. The samples were amplified using a thermal cycler ABI PRISM 7000 Sequence Detection System (Applied Biosystems, Foster City, CA). The parasite loads were estimated by using a standard curve constructed with DNA obtained from trypomastigote cultures of *T. cruzi*.

### CK-MB and ALT assays

Levels of the CK-MB, one of the markers of myocardial injury, were determined in the sera from infected mice (15 days postinfection) using a commercial CK-MB test (Labtest REF 118.3/1). A 0.05 mL aliquot of the serum or a calibrator was incubated at 37^°^C for 5 min. The absorbance (A) was read twice using a 340 nm filter to obtain the ΔA for the sample. The results were expressed as the activity of CK-MB (U/L) = ΔA x calibration factor. The activity of the alanine aminotransferase (ALT) was measured using a commercial Labtest Kit (ref: 53MS100090127) according to the manufacturer's instructions.

### Statistical analyses

The data obtained from *in vivo* experiments are expressed as the mean ± SEM. For statistical analyses, ANOVA was used, followed by a Bonferroni posttest analysis. For comparison of two groups, Student's t test was used. Survival curves were analyzed using the Kaplan-Meier method. All analyses were performed using Prism 5.0 software (GraphPad, San Diego, CA).

## Results

### Inbred mouse strains vary in their susceptibility to infection with the Y strain of *T. cruzi in vivo*

**Figure 1:**
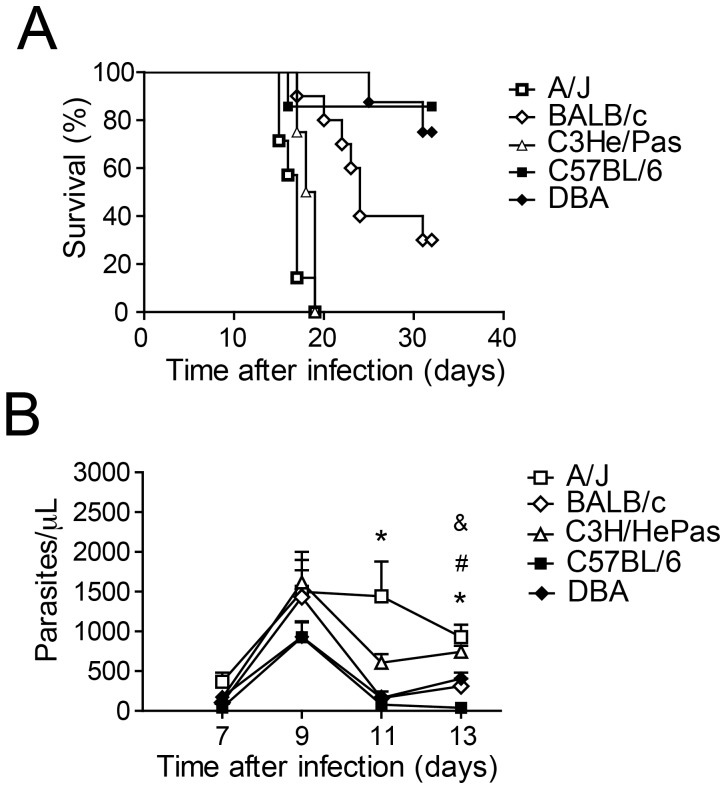
Inbred mouse strains vary in their susceptibility to infection by the Y strain of *T. cruzi*. Male mice were infected i.p. with 1000 trypomastigotes when eight weeks old and included A/J (n = 8), BALB/c (n = 10), C3H/HePas (n = 8), C57BL/6 (n = 7) and DBA (n = 8) strains. (**A**) Mortality was evaluated by daily inspection of the cages and differed (*P*<0.05) between BALB/c and C57BL/6, BALB/c and DBA, BALB/c and A/J, BALB/c and C3H/HePas, C57BL/6 and A/J, C57BL/6 and C3H/HePas, DBA and A/J, and DBA and C3H/HePas. Data are representative of those found in three independent experiments. (**B**) Parasitemia was quantified microscopically by counting the parasites in 5 μl of citrated blood obtained from the tail lateral vein at days 7, 9, 11 and 13 days postinfection. At day 11, (*) indicates *P*<0.05 between A/J and all other groups. At day 13, (*) indicates *P*<0.05 between A/J and BALB/c, A/J and C57BL/6, and A/J and DBA; (#) indicates *P*<0.05 between C3H/HePas and BALB/c, C3H/HePas and C57BL/6, and C3H/HePas and DBA; and (&) indicates *P*<0.05 between DBA and C57BL/6. Data are representative of those found in three independent experiments.

Although inbred mouse strains that differ in their susceptibility to a specific pathogen are an extremely useful tool for the identification of the relevant genes, little has been learned regarding the resistance to Chagas disease using this approach. Therefore, we initially infected distinct inbred mouse strains with the Y strain of *T. cruzi* to detect any differences in their susceptibility to the pathogen, as measured by blood parasitemia and mouse mortality. The results, presented in **Figure 1** **,** highlight the considerable variation in the susceptibility to acute infection among the A/J, BALB/c, C3H/HePas, C57BL/6 and DBA strains of mice. The C57BL/6 and DBA mice presented the highest survival rates, whereas the A/J and C3H/HePas mice presented higher mortality (**Fig. 1A**). The analysis of parasitemia on days 7, 9, 11, and 13 postinfection revealed that the A/J animals had a high peak count of parasites in the blood and maintained a higher number of circulating parasites in the blood, as illustrated by the high parasitemia of A/J mice at day 11 postinfection (**Fig. 1B**). The blood of the C57BL/6 mice had a low peak count of parasites and, after 13 days of infection, very few circulating parasites remained. Altogether, our data indicate that the A/J and C57BL/6 strains were the most different regarding host resistance. These features have been previously described for *T. cruzi* strains and, together with our data, emphasize the relevance of studying these two mouse strains to assess the host genetic factors contributing to the control of infection [27], [28], [29], [30].

### Macrophages from C57BL/6 and A/J vary in their susceptibility to *T. cruzi* infection in vitro

**Figure 2:**
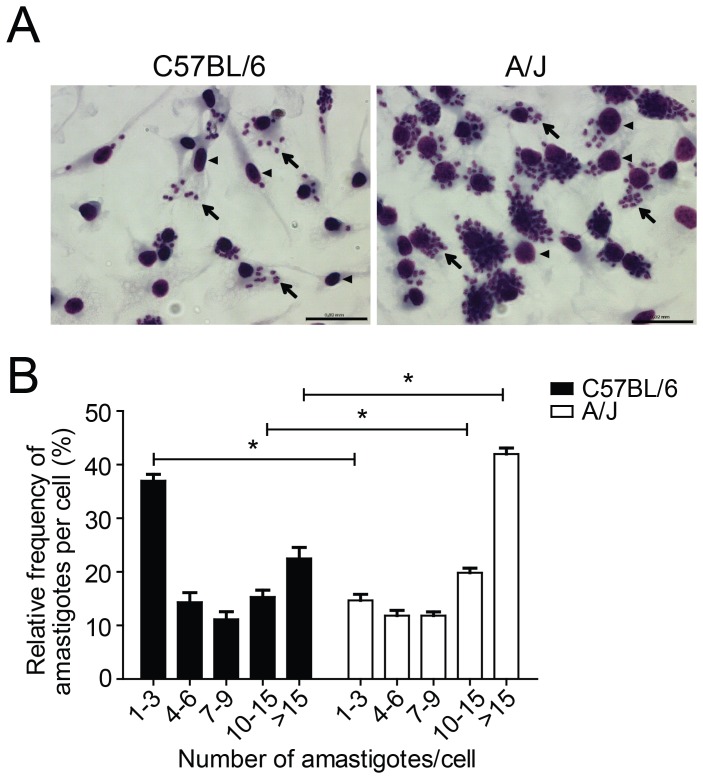
Macrophages from A/J mice are more susceptible to *T. cruzi* infection than those from C57BL/6 mice. Bone marrow-derived macrophages (BMDMs) from A/J and C57BL/6 mice were infected at a multiplicity of infection of 5 with trypomastigotes of the Y strain of *T. cruzi*. At 48 h postinfection, cells were fixed and stained with Giemsa. (**A**) Representative micrographs of infected cells. Arrows indicate the intracellular amastigotes; arrowheads indicate the macrophage nuclei. The scale bar represents 20 µm. (**B**) Percentage of parasite-infected BMDMs containing 1 to 3, 4 to 6, 7 to 9, 10 to 15, or >15 amastigotes per cell. A total of 100 infected cells were scored in each of the 6 distinguished replicates. Data are expressed as the mean ± standard deviation of the 6 replicates. The experiment shown is representative of those found in three independent experiments. (*) indicates *P*<0.05.

The early manifestations of *T. cruzi* infection in humans and in experimental models of infection support an important role of innate immunity for successfully controlling the initial replication of the parasites. Macrophages are an important host cell for the outcome of *T. cruzi* infection; therefore, strain differences in the capacity to control the initial phase of infection could involve differences in the capacity of these cells to restrict the multiplication of the parasites. To verify whether genetic differences between the C57BL/6 and A/J strains influence the effectiveness of their innate immune responses to *T. cruzi*, we evaluated parasite multiplication in BMDMs obtained from these two mouse strains. The BMDMs obtained from the A/J and C57BL/6 mice were infected with the trypomastigote form of *T. cruzi* at an MOI of 5. After infection for 48 h, the intracellular amastigotes were stained and their quantity microscopically scored. We found that the number of amastigote parasites within the cytoplasm of the infected BMDMs differed greatly between these two mouse strains (**Fig. 2A**). The A/J-derived BMDMs contained higher parasite loads, with an average of 10.1±1.2 amastigotes per cell. In contrast, the BMDMs from the C57BL/6 mice averaged only 5.8±0.8 amastigotes per cell. The internalization of the trypomastigote form of *T. cruzi* by the BMDMs from these two mice strains was similar, as assessed by estimating the number of intracellular parasites after infection for 4 h (data not shown). Therefore, the data shown in Fig. 2 suggest that intrinsic genetic differences between these two strains play a role in their ability to restrict the intracellular replication of *T. cruzi*.

### C57BL/6 and A/J mice present distinct histopathology during experimental *T. cruzi* infection

**Figure 3:**
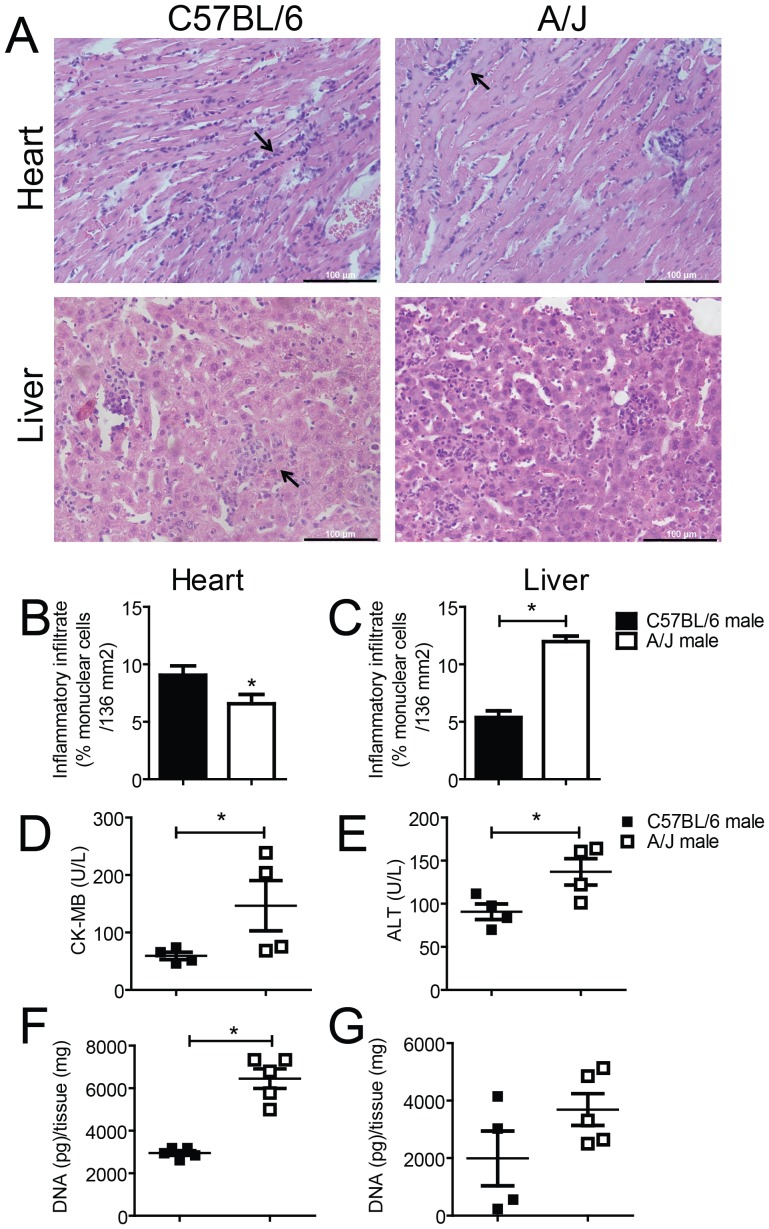
C57BL/6 and A/J mice infected with *T. cruzi* differ in cardiac and hepatic histopathology, tissue injury and parasite load. A/J and C57BL/6 mice were infected i.p. with 1000 trypomastigotes of the Y strain of *T. cruzi*. Infected animals were euthanized 15 days postinfection, and cardiac muscle and liver were collected and processed as described in Material and Methods. (A) Tissue samples were stained with hematoxylin-eosin to determine the presence of inflammatory infiltrates. Representative micrographs of C57BL/6 and A/J tissues are shown. Arrows indicate the presence of infiltrates of mononuclear cells in the myocardium and among the hepatocytes; the scale bar represents 100 µm. (B–C) Inflammatory infiltrates in the heart (B) and liver (C) were determined by counting nuclei of infiltrating cells after staining the organ sections with hematoxylin-eosin. (D–E) Lesions in the heart (D) and liver (E) of infected mice were estimated by quantifying the levels of creatine kinase enzyme (CK-MB) and alanine transaminase (ALT), respectively. (F–G) Parasite loads in the heart (F) and liver (G) of infected mice were determined by quantitative real time PCR using specific primers for *T. cruzi* (as described in Material and Methods). Data are representative of those found in three independent experiments. (*) indicates *P*<0.05.

Variability in the pattern of tissue invasion by *T. cruzi* parasites in infected individuals is often observed in Chagas disease. Likewise, previous reports showed that genetic variation between mice strains was influenced by tissue tropism in chronic models of experimental infection by a single strain of *T. cruzi* [30]. Because our initial data indicated much variation among mouse strains in the parasitemia induced by *T. cruzi*, we investigated the replication of the parasite, the inflammatory response and the extent of the tissue damage in the hearts and livers of the infected mice. Examination of the tissue sections 15 days postinfection revealed distinct patterns of inflammatory infiltration between the A/J and C57BL/6 mice. As observed in **Fig. 3A**, the hearts of the infected C57BL/6 mice were more highly infiltrated by inflammatory cells than were those of the infected A/J mice. By contrast, the hepatic tissue of the A/J mice showed a higher inflammatory infiltrate than did that of the C57BL/6 mice (**Fig. 3A**). These phenotypes were further confirmed by quantifying the number of cells in the inflammatory infiltrate in these organs (**Fig. 3B, C** **)**. To evaluate the extent of the tissue damage, we quantified the activity of the serological enzymatic markers of tissue injury: cardiac creatine kinase (CK-MB) and hepatic alanine aminotransferase (ALT). Our data showed that the CK-MB and ALT activities were higher in the tissue samples from the A/J mice compared with the C57BL/6 mice (**Fig. 3D, E**). Importantly, the accurate estimation of the extent of the organ lesions by the organ-specific enzyme activity analysis suggests that the A/J mice suffered worse damage to both organs despite their reduced cardiac inflammatory infiltrate. These findings support the hypothesis that the cardiac lesion induced by *T. cruzi* more likely resulted from tissue destruction induced by parasite replication than from damage triggered by the inflammatory infiltrate itself. Importantly, the reduced cardiac inflammatory response of the A/J mice coincided with a higher cardiac burden of *T. cruzi* than was observed in the C57BL/6 mice (**Fig. 3F**). We also observed a tendency for a higher hepatic parasite burden in the infected A/J mice compared with their C57BL/6 counterparts (**Fig. 3G**). Collectively, these data indicate that the C57BL/6 mice differ from the A/J mice not only in resistance to lethal infection and parasitemia, but also in tissue lesion, inflammatory response and parasite burden in the organs. These features support further investigation of these two inbred mouse strains to determine the genetic basis for the immune response that leads to resistance to infection.

### Male and female offspring from an A/J x C57BL/6 cross differ in their susceptibility to experimental *T. cruzi* infection

**Figure 4:**
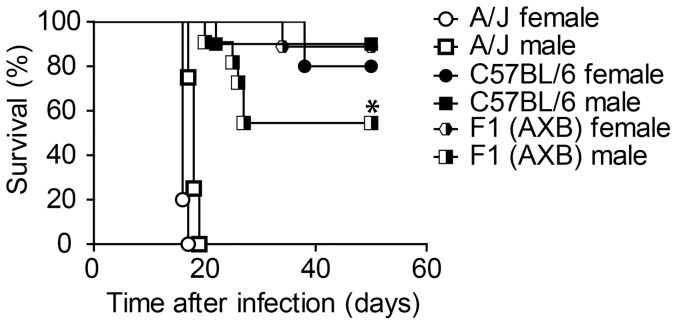
Gender influences susceptibility to infection in F1 offspring of A/J females crossed with C57BL/6 males. Age-matched males and females of parental A/J and C57BL/6 mouse strains together with heterozygous F1 offspring (F1(AXB)) of A/J females crossed with C57BL/6 males were infected i.p. with 1000 trypomastigotes of the Y strain of *T. cruzi*. Mouse numbers were as follows: A/J male (n = 11), A/J female (n = 8), C57BL/6 male (n = 11) C57BL/6 female (n = 8), F1(AXB) male (n = 9), F1(AXB) female (n = 11). Mortality was evaluated by daily inspection of the cages. Data are representative of those found in seven independent experiments. (*) indicates *P*<0.05 in comparisons between F1(AXB) males and the other experimental groups.

A genetic approach to the identification of traits that contribute to a specific resistant phenotype demands a thorough understanding of the pattern of inheritance for the phenotype. To investigate the genetic source of resistance variation to *T. cruzi* infection by the C57BL/6 and A/J mice, we established a heterozygous F1 population (F1(AXB)) by breeding A/J females with C57BL/6 males. Females and males of the F1(AXB) population were infected together with controls of the C57BL/6 and A/J parental strains. The mortality was evaluated over a period of 50 days. As expected, the A/J male and female mice were highly susceptible and succumbed at day 18–20 after infection, whereas both sexes of the parental C57BL/6 strain were resistant (**Fig. 4**). Males of the F1(AXB) generation showed mortality intermediate between the parental strains; however, F1(AXB) females were resistant, suggesting dominance of the C57BL/6 resistance trait.

### A parent-of-origin effect determines sex-related differences among F1 offspring from A/J x C57BL/6 crosses

**Figure 5:**
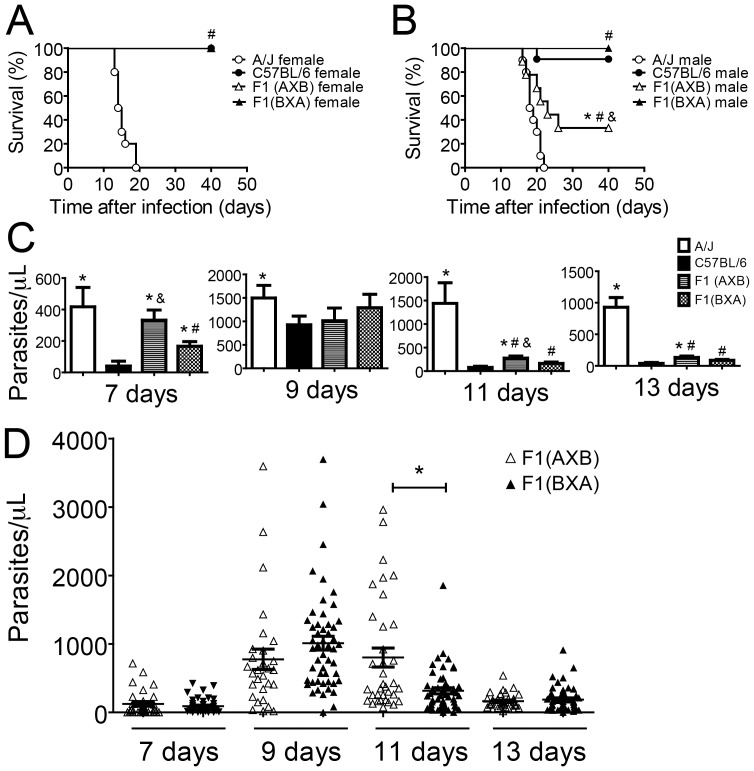
Direction of the cross between A/J and C57BL/6 mice determines gender differences in the susceptibility to infection of the F1 offspring. Age-matched A/J, C57BL/6, F1(AXB) (offspring of A/J females and C57BL/6 males) and F1(BXA) (offspring of C57BL/6 females and A/J males) mice were infected i.p. with 1000 trypomastigotes of the Y strain of *T. cruzi*. Mortality of female (A) and male (B) mice was evaluated by daily inspection of the cages. Mouse numbers were as follows: A/J male (n = 10), A/J female (n = 10), C57BL/6 male (n = 11), C57BL/6 female (n = 08), F1(AXB) male (n = 08), F1(AXB) female (n = 11), F1(BXA) male (n = 7) and F1(BXA) female (n = 8). (C) Parasitemia of male mice was quantified by microscopically counting the parasites in 5 μl of citrated blood obtained from the tail lateral vein on days 7, 9, 11 and 13 post-infection. Data shown is one representative experiment of those found in three independent experiments. (*), (#), and (&) indicate *P*<0.05 in relation to C57BL/6, A/J and F1(BXA) respectively. (D) Parasitemia of F1(AXB) (open triangle) and F1 (BXA) (closed triangle) male mice on days 7, 9, 11, 13 post-infection. The data shown is a pool of 8 independent experiments; each plotted symbol represents the parasitemia of a single mouse. (*) indicates *P*<0.05.

The data presented so far show that F1(AXB) males differ from F1(AXB) females in resistance to infection with *T. cruzi*, although the difference in resistance between the infected males and females within both parental strains was small and statistically insignificant (**Fig. 4**). These data suggest that sex-related physiological differences (such as hormonal influences) could not explain the difference in susceptibility of the sexes in the F1 generation. To further evaluate the sex-related differences in resistance seen in the F1(AXB) mice, we generated additional F1 progeny by breeding female C57BL/6 mice with male A/J mice (F1(BXA)). As show in **Table 1**, the direction of the cross between the parental A/J and C57BL/6 mice changed the composition of the sex chromosomes in the F1 generation only in the males (**Table 1**). This feature supports a role of the sex chromosomes in host resistance. We thus performed an experiment in which we simultaneously infected F1(AXB) and F1(BXA) females and males together with controls of the parental C57BL/6 and A/J strains and measured survival and parasite loads in the blood of the infected animals. We observed that F1 females from both types of cross (F1(AXB) and F1(BXA)) were as resistant to infection as the C57BL/6 parental females, supporting the conclusion that resistance to infection is a dominant trait (**Fig. 5A**). By contrast, the F1(BXA) males were as resistant as the C57BL/6 mice, whereas the F1(AXB) males showed a mortality rate intermediate between the two parental strains (**Fig**. **5B**). The parasitemia of male siblings from both crossings was also compared 7, 9, 11 and 13 days after infection. Our results indicate that at days 7 and 11 after infection, the parasitemia of the F1(AXB) males was significantly higher as compared to parasitemia of F1(BXA) (**Fig. 5C** **)**. To further evaluate the increased parasitemia in males of the F1(AXB) as compared to F1(BXA) we analyzed the parasitemia of infected F1 mice obtained in eight experiments performed independently. By pooling together data from different experiments, we found statistically significant differences in the parasitemia of F1(AXB) as compared to F1(BXA) only at day 11 post infection **(** **Fig. 5D** **)**. Importantly, at day 11, we detected the higher differences in parasitemia of A/J as compared to C57BL/6 strain **(** **Fig. 1B** **)**. Nonetheless, the results shown in Fig. 5D illustrate that parasite levels in the blood of mice infected with the Y strain of *T. cruzi* is highly variable, thus suggesting that parasitemia is not the best parameter to evaluate host resistance to Y strain of *T. cruzi*.

**Table 1:** Strain of origin of the X and Y chromosomes in F1 offspring from reciprocal crosses between A/J x C57BL/6 parental mice.

Sex chromosomes^a^			
F1 population	Resistant parent	F1 male	F1 female
F1(AXB)^b^	Father	X^AJ^ + Y^B6^	X^AJ^ + X^B6^
F1(BXA)^c^	Mother	X^B6^ + Y^AJ^	X^B6^ + X^AJ^

a AJ indicates origin from the A/J strain; B6 indicates origin from the C57BL/6 strain. ^b^Mother A/J, father C57BL/6. ^c^Mother C57BL/6, father A/J.

**Figure 6:**
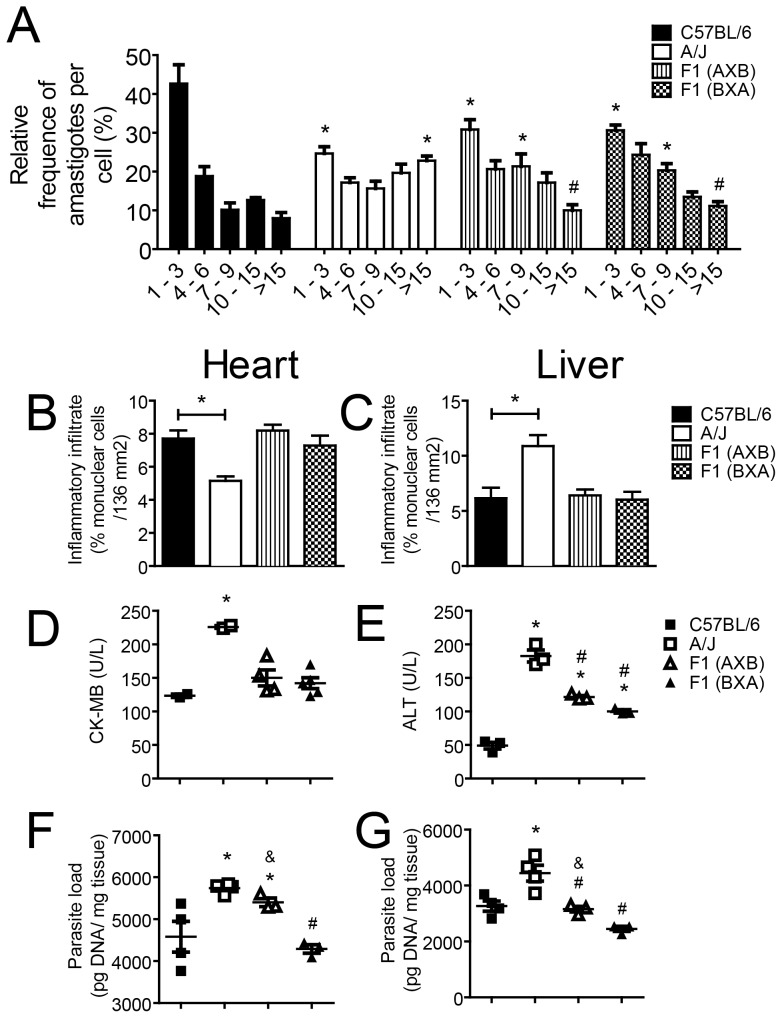
Analysis of macrophage infection, histopatology and parasite load of F1(AXB) and F1(BXA) male mice infected with *T. cruzi*. (A) Bone marrow-derived macrophages (BMDMs) from A/J, C57BL/6, F1(AXB) and F1(BXA) mice were infected at a multiplicity of infection of 5 with trypomastigotes of the Y strain of *T. cruzi*. At 48 h postinfection, cells were fixed and stained with Giemsa for determination of the percentage of parasite-infected BMDMs containing 1 to 3, 4 to 6, 7 to 9, 10 to 15, or >15 amastigotes per cell. A total of 100 infected cells were scored in each of the 6 distinguished replicates. Data are expressed as the mean ± standard deviation of the 6 replicates. (*) and (#) indicate *P*<0.05 in relation to C57BL/6 and A/J BMDMs, respectively. (B–G) A/J, C57BL/6, F1(AXB) and F1(BXA) male mice were infected i.p with 1000 trypomastigotes of the Y strain of *T. cruzi*. Infected mice were euthanized 15 days postinfection, and cardiac muscle and liver were collected and processed as described in Material and Methods. (B–C) Tissue samples were stained with hematoxylin-eosin (H&E) for determination of inflammatory infiltrates in the heart (B) and liver (C). (D–E) Lesions in the heart (D) and liver (E) of infected mice were estimated by quantifying the levels of creatine kinase enzyme (CK-MB) and alanine transaminase (ALT), respectively. (F–G) Parasite loads in the heart (F) and liver (G) of infected mice were determined by quantitative real time PCR using specific primers for *T.cruzi* (as described in Material and Methods). (B–G) (*), (#), and (&) indicate *P*<0.05 in relation to C57BL/6, A/J and F1(BXA) respectively.

To investigate if the increased susceptibility of F1(AXB) mice was a feature associated with macrophages resistance to infection, we determined the intracellular replication of *T. cruzi* in BMDMs obtained from these strains. BMDMs were generated from C57BL/6, A/J, F1(AXB) and F1(BXA) male mice and infected with *T. cruzi* for 48 hours to evaluate intracellular parasite replication. As previously demonstrated in Fig. 2, BMDM obtained from A/J were significantly more permissive to *T. cruzi* replication as compared to C57BL/6 **(** **Fig. 6A** **)**. Moreover, both F1 crossings showed an intermediate phenotype between C57BL/6 and A/J **(** **Fig. 6A** **)**. Of note, we found no significant differences between BMDMs from F1(AXB) and F1(BXA) for intracellular parasite replication, indicating that the increased susceptibility of the F1(AXB) mice is not macrophage associated **(** **Fig. 6A** **)**. Thus, additional *loci* independent of those contained in the sex chromosomes may be involved in the macrophage resistance to *T. cruzi* infection. To further characterize the increased susceptibility of the F1(AXB) mice, we evaluated additional parameters related to *T. cruzi* infection *in vivo*: the replication of the parasite in the target organs, the inflammatory response and the extent of the tissue damage in the heart and liver. Examination of the inflammatory infiltrate in the heart and liver of infected mice showed that the F1(AXB) and F1(BXA) did not differ between each other and from the pattern observed in the organs of C57BL/6 mice **(** **Fig. 6B and 6C** **)**. Analysis of the activity of the enzymes CK-MB and ALT indicated that tissue lesion in the heart and the liver of F1(AXB) and F1(BXA) were not statistically different **(** **Fig. 6D and 6E** **)**. However, by quantifying parasite DNA in the heart and liver, we found an increased parasite load in the organs of the F1(AXB) as compared to F1(BXA) **(** **Fig. 6F and 6G** **)**. Of note, the parasite load detected in the heart of F1(AXB) mice was not statistically different from that found in A/J parental strain **(** **Fig. 6F** **).** These observations support our findings related to the increased susceptibility of F1(AXB) mice compared with the F1(BXA) male mice.

## Discussion

The development of Chagas disease is determined by a complex interaction between the genetic traits of both the *T. cruzi* parasite and the infected host. In this detailed study, we have explored differences in the susceptibility to infection between two genetically distinct mouse strains to gain insight into the role of host genetics in resistance to Chagas disease. By employing a mouse model of acute and lethal *T. cruzi* infection, we showed that the higher mortality of the A/J mice compared with the C57BL/6 strain correlates with additional characteristics of the infection. The A/J mice presented an uncontrolled parasitemia during the initial stages of infection and a characteristic histopathology that was associated with a higher parasite burden and more extensive tissue lesions. The strikingly opposite features of the *T. cruzi* infections in these two mouse strains corroborate the relevance of this model for investigation of the role of host genetics in host resistance against *T. cruzi* infection. Most importantly, our approach using a non-informative F1 population derived from the directional intercross between the A/J and C57BL/6 strains revealed a parent-of-origin effect in the susceptibility to *T. cruzi*. This particular finding suggests that one or more *loci* on the murine chromosome X contribute to the control of infection and resistance to Chagas disease. Importantly, previous studies performed by Minoprio and collaborators, have demonstrated that BALB.Xid mice, which is defective in Bruton's tyrosine kinase gene (*Btk*), and are a model of human X-linked immunodeficiency, are defective to generate B cell response to *T. cruzi* [31]. Interestingly, the crossing of BALB.Xid females with BALB/c males resulted in F1 male mice that phenocopy the BALB.Xid, whereas the F1 female were similar to BALB/c [31]. Further studies revealed that *Xid* mediated resistance to *T. cruzi* infection was IFN-γ dependent [32]. In this context, although we detected no differences in production and responses to IFN-γ between F1(AXB) x F1(BXA) (data not shown), further investigation will be required to address if the putative X-linked mutations carried by A/J mouse maps on the *Xid locus*.

The resistance in the females obtained from the intercross of C57BL/6 and A/J was identified as a dominant trait. The presence of a single A/J-derived X chromosome in the F1(AXB) males suggested that the X chromosome might be important to genetic control of murine infection by *T. cruzi*. Importantly, the A/J mice were more susceptible than both the F1(AXB) and F1(BXA) offspring, as revealed by their greater parasitemia, increased parasite loads in heart and liver and higher mortality rate during infection, which suggested that additional *loci* regulate host resistance to *T. cruzi* infection. Altogether, these results indicate that even though the murine X chromosome may carry resistance genes that function improperly in the A/J strain, the resistance trait encoded on the X chromosome possesses an incomplete penetrance. Thus, multiple host *loci* besides the one suggested here on chromosome X might regulate the host resistance to Chagas disease. This hypothesis is also supported by experiments shown in **Fig. 6**. For instance, the demonstration that inflammatory infiltrate and tissue lesion found in the heart and in the liver of the F1(AXB) and F1(BXA) mice did not differed between each other and from those found in C57BL/6 mice, support the hypothesis that inflammatory response in these two mice strains is a dominant trait that is independent of the parent of origin effect on X chromosome.

Host genetic traits that are determined or influenced by gender have been previously identified in diverse murine models of infective diseases [33], [34], [35], [36]. For protozoan parasites such as *Plasmodium chabaudi,* sex-biased hormonal differences are an important factor influencing host susceptibility [37], [38], [39]. Similarly, the influence of sex on human susceptibility to *T. cruzi* has also been reported [40], [41]. However, it is not clear if this susceptibility is associated with sex-biased hormonal differences or with immune-response genes that are encoded on the sex chromosomes. Importantly, the pattern of inheritance in reciprocal intercrosses between inbred mouse strains is very useful to identify the involvement of a parent-of-origin effect controlling a given trait, such as X- or Y-chromosome polymorphism or genomic imprinting. Of note, the genetic-based approaches to understanding the pathogenesis of *T. congolense* have indicated that a major host *locus* contributing to infection control is modulated by imprinting patterns determined by a parent-of-origin effect [13]. However, the *locus* previously identified in a model of *T. congolense* infection is not the same as that operating in the murine model of *T. cruzi* infection employed here. The genetic-based approaches to understanding the pathogenesis of *T. congolense* have indicated that the host *loci* contributing to infection control are effectively modulated by imprinting patterns [13], which may not the case in our study. As demonstrated in Figs. 4 and 5, we found no difference in survival between males and females within either parental strain infected with *T. cruzi*, thus suggesting that a hormonal influence by sex and imprinting patterns determined by a parent-of-origin effect could not explain the sex-based difference in susceptibility reported for the F1 generation. However, future studies with different parasite strains and infection dose will be required to further evaluate the hormonal influence by sex and imprinting patterns determined by a parent-of-origin effect in the susceptibility of C57BL/6 and A/J mouse strains to *T. cruzi* infection. Nonetheless, our data support a role of the X chromosomes in host resistance; a feature reinforced by the reciprocal-cross experiments performed with the A/J and C57BL/6 parental strains. As indicated in Table 1, the distribution of sexual chromosomes in F1 male individuals may reveal a role of these chromosomes in host resistance. Specifically, our data indicated that the female offspring from both reciprocal crosses (F1(AXB) and F1(BXA) females) were as resistant to infection as the C57BL/6 parental females, supporting the conclusion that resistance to infection is a dominant trait. Furthermore, based on the analysis of the F1 male populations, we favor a role for the X chromosomes in host resistance. In this scenario, the reduced resistance of the F1(AXB) males compared with the F1(BXA) females possibly results from the inheritance of susceptible alleles present on the X chromosome derived from the parental A/J female. According to this hypothesis, the Y chromosome transmitted to the F1(AXB) males from the male C57BL/6 parents has little or no role in the resistance to *T. cruzi* infection. The data provided here encourage the use of the A/J and C57BL/6 mouse models of infection by the Y strain of *T. cruzi* to map other *loci* and genes that regulate host resistance and are located on the X chromosome. Because little is known regarding mammalian forward genetic screening using neglected parasites such as *T. cruzi*, this approach may reveal novel host mechanisms that function to resist and fight infectious diseases.

It is well established that macrophages are essential for controlling *T. cruzi* infections, playing a lytic role against parasites either when activated by innate immune cells or by an adaptive immune response. Here, we demonstrated that BMDMs obtained from the A/J mice are less able to restrict the intracellular replication of *T. cruzi* amastigotes than are the similarly derived BMDMs from the C57BL/6 mice. Given that the life cycle of *T. cruzi* within the mammalian host is mostly intracellular, it is reasonable to hypothesize that the resistance mechanisms may, at least partially, rely on cellular mediated mechanisms to restrict intracellular parasite replication. These observations are supported by several independent reports that associate increased parasite replication in macrophages with increased susceptibility to infection in a murine model of experimental infection [24], [42], [43], [44], [45]. However, our results obtained from BMDMs of both intercrosses showed that F1(AXB) and F1(BXA) possess a similar intermediate resistance **(** **Fig. 6A** **)**. Therefore, this putative resistance *loci* contained in chromosome X investigated herein may not participate in the regulation of innate immune responses by the macrophage to restrict the infection by *T. cruzi*. Nonetheless, additional studies addressing the mechanisms of resistance of macrophages from the intercross of C57BL/6 and A/J may facilitate the identification of important *loci* regulating innate immunity and effector mechanisms in the host resistance to *T. cruzi* infection.

Regardless to future investigation of resistance of BMDMs from the crossing of A/J and C57BL/6 mouse, our data presented herein with the so called “non informative F1 population” support the existence of specific genes on the mouse chromosome X that account for host resistance to lethality of *T. cruzi* infections. Our findings indicating an importance of the X chromosome for host resistance may support the future identification of the relevant alleles and the specific genes contained in this putative *locus*. Identification of the relevant genes may contribute not only to our understanding of how host immunity fights infectious diseases but also to the identification of novel therapeutic targets that may be modulated to treat Chagas disease and other important protozoan diseases in humans.
